# Invasive urothelial carcinoma, lymphoma-like/plasmacytoid variant, successfully treated by radical cystectomy with adjuvant chemotherapy: a case report

**DOI:** 10.1186/s13256-016-0806-x

**Published:** 2016-03-08

**Authors:** Mari Ohtaka, Takashi Kawahara, Yohei Kumano, Yoko Maeda, Takuya Kondo, Taku Mochizuki, Hiroaki Ishida, Yusuke Hattori, Jun-ichi Teranishi, Yasuhide Miyoshi, Yasushi Yumura, Masahiro Yao, Yoshiaki Inayama, Hiroji Uemura

**Affiliations:** 1 Department of Urology and Renal Transplantation, Yokohama City University Medical Center, 4-57 Urafune-cho Minami-ku, Yokohama, 232-0024 Japan; 2 Department of Urology, Yokohama City University Graduate School of Medicine, 4-57 Urafune-cho Minami-ku, Yokohama, 232-0024 Japan; 3 Department of Diagnostic Pathology, Yokohama City University Medical Center, 4-57 Urafune-cho Minami-ku, Yokohama, 232-0024 Japan

**Keywords:** Urothelial carcinoma, Plasmacytoid variant, Gemcitabine, Cisplatin, Radical cystectomy

## Abstract

**Background:**

Invasive urothelial carcinoma, lymphoma-like/plasmacytoid variant, is a rare histological type of bladder cancer similar to plasma cells and is an aggressive variant of urothelial carcinoma associated with a poor prognosis.

**Case presentation:**

A 41-year-old Asian man was referred to our hospital due to macroscopic hematuria. Cystoscopy detected a non-papillary tumor, and a transurethral resection of the bladder tumor revealed pT1N0M0 bladder cancer. A pathological examination showed high-grade invasive urothelial carcinoma and a component of signet ring cell carcinoma. A follow-up of the transurethral resection with radical cystectomy was carried out, and a pathological examination showed infiltrating urothelial carcinoma, with partial features of the plasmacytoid variant. We added chemotherapy treatment with gemcitabine and cisplatin for two cycles. Our patient has been free from recurrence for 2 years.

**Conclusions:**

We herein report the case of a patient with a plasmacytoid variant of urothelial carcinoma controlled with radial cystectomy and subsequent chemotherapy.

## Background

Invasive urothelial carcinoma, lymphoma-like/plasmacytoid variant (PUC), is a rare histological type of bladder cancer similar to plasma cells and is an aggressive variant of urothelial carcinoma (UC) associated with a poor prognosis. The first report for PUC was provided by Sahin *et al.*; to date, only approximately 100 cases of PUC have been reported in the English literature [1–5]. We herein report a case of a patient with plasmacytoid urothelial carcinoma that was successfully controlled with chemotherapy following radical cystectomy.

## Case presentation

A 41-year-old Asian man was referred to our hospital due to macroscopic hematuria. His past history included middle ear cholesteatoma and he had no smoking history. Cystoscopy detected a non-papillary tumor, and a transurethral resection of the bladder tumor (TUR-Bt) and a magnetic resonance imaging (MRI) scan revealed cT3N0M0 bladder cancer.

### Laboratory data at the time of admission

The hematological and biochemical data showed no abnormal findings. A urinary analysis resulted in the following: pH 6.5, red blood cell count 10–19/high-power field (HPF), white blood cell count 1–4/HPF, protein negative, and glucose negative. The urinary cytology was class III.

### Imaging findings

**Fig. 1:**
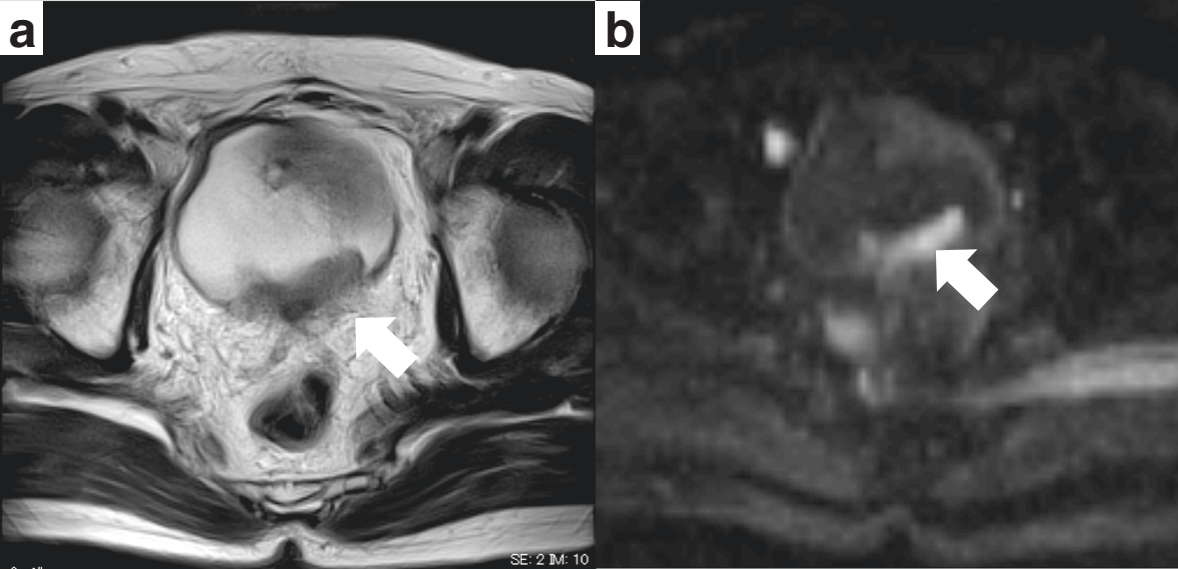
Magnetic resonance imaging findings in (**a**) T2-weighted and (**b**) diffusion-weighted images. The mass was believed to be invasive outside of the bladder wall (*arrow*)

An MRI scan showed that the tumor extended over the bladder, as seen in Fig. 1. No obvious distal and local lymphadenopathy was observed on a non-contrast computed tomography (CT) scan.

### Operative procedure

**Fig. 2:**
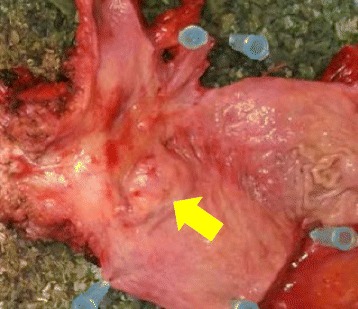
A surgical specimen from the radical cystectomy. The tumor was located in the bladder trigone (*arrow*)

Our patient underwent a TUR-Bt, and a pathological examination revealed urothelial carcinoma (high-grade, pT1, G2) and signet ring cell carcinoma was found in a portion of the bladder tumor. Two months after the TUR-Bt, a radical cystectomy was performed according to the tumor grade of malignancy and the imaging findings (Fig. 2).

### Pathological findings

**Fig. 3:**
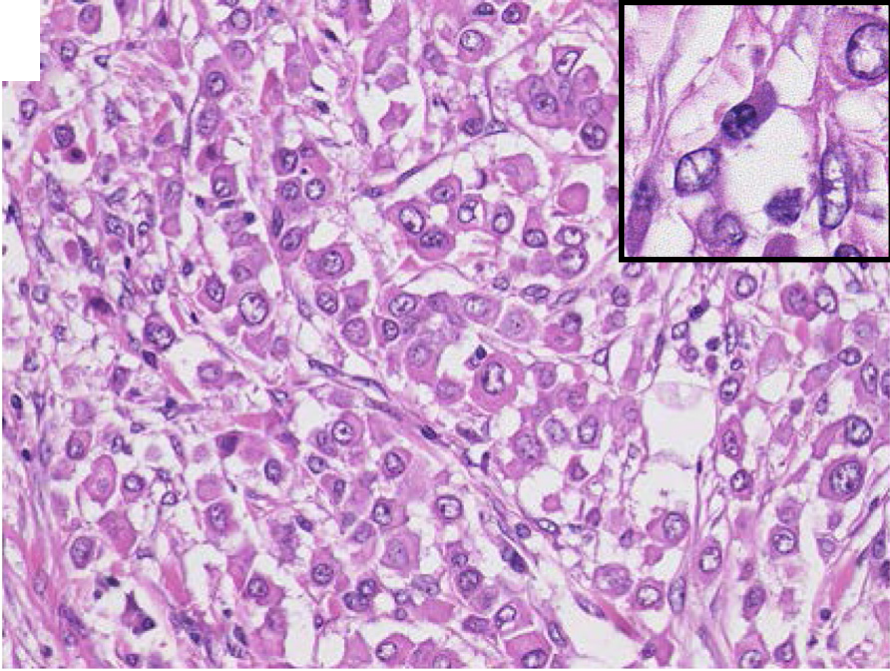
Hematoxylin and eosin stain. Plasmacytoid urothelial carcinoma was characterized by isolated cells with uneven nuclear distribution. Invasive plasma cell was founded sparsely (*enlarged part of image*)

Grossly, the infiltrating neoplasm, measuring 25 × 20 mm in size, was located in the bladder trigone. The pathological stage was pT2bN0M0. Histologically, the neoplasm was composed of isolated atypical cells that had relatively abundant eosinophilic cytoplasm with eccentric nuclei, showing moderate pleomorphism. Perinuclear pale regions were occasionally seen. These features partly resembled plasma cells (Fig. 3). The immunohistochemical profile of the tumor cells was positive for keratin CAM5.2 and CK20, but negative for CK7, CD56, chromogranin A, synaptophysin, CD20, CD79a, kappa and lambda. Thus, the diagnosis of infiltrating high-grade urothelial carcinoma, plasmacytoid variant, was made. Plasma cells have a similar form to signet ring cells, thus it is difficult to distinguish these cells. However, plasma cells frequently show CD138 positivity, which may be useful for discriminating between the two types of cells.

### Immunohistochemistry for PD-1 and PD-L1

**Fig. 4:**
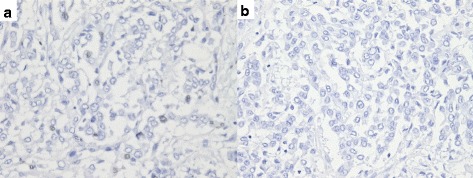
Immunohistochemistry for PD-1 (**a**) and PD-L1 (**b**) expression. There was no positive expression in both PD-1 and PD-L1

An immunohistochemical analysis was performed on 5-μm-thick sections. The slides were dewaxed with xylene and hydrated with gradient ethanol, and microwaved at the high level for 2 minutes and then at the medium-low level for 30 minutes for heat antigen retrieval (Target Retrieval Solution pH 9, Dako, Carpinteria, CA, USA). After 3 % hydrogen peroxidase blocking, the samples were incubated overnight at 4 °C with a primary antibody to PD-1 (dilution 1:50, Santa Cruz Biotechnology, Santa Cruz, CA, USA) or PD-L1 (dilution 1:50, Santa Cruz Biotechnology). The slides were then treated with a broad-spectrum secondary antibody (Invitrogen, Grand Island, NY, USA) and washed (Envision FLEX Wash Buffer, Dako). After diaminobenzidine staining, the slides were counterstained with hematoxylin, dehydrated with gradient ethanol and xylene, and then sealed. There were no positive finding (Fig. 4).

### Postoperative course

Two months after the total cystectomy, adjuvant chemotherapy with gemcitabine and cisplatin was administered (G: 2,000 mg/m^2^, C: 63 mg/m^2^). We used the following dosing schedule: day 1 G: 1,000 mg/m^2^, day 2 C: 63 mg/m^2^, and day 8 G: 1,000 mg/m^2^ that was administered in two cycles. No adverse event was observed. We followed up our patient with cytology and a CT scan every 3 months. Our patient has not experienced any recurrence of bladder cancer for 2 years following the radical cystectomy.

## Discussion

PUC is a rare histological type of bladder cancer similar to plasma cells and is an aggressive variant of UC associated with a poor prognosis [6]. The first case of PUC was reported by Sahin *et al*.; to date, only approximately100 cases of PUC have been reported in the English literature [1–3, 5]. Most patients were in their 60s; the male-to-female ratio was 9:1. This variant is typically diagnosed at an advanced pathological stage (64 % pT3, 23 % pT4), showing metastases in 60 % of the patients [3]. This tumor initially presents as a high-grade, high-stage lesion, and diffusely invades the bladder wall; however, patients have no specific symptoms, which can lead to a delayed diagnosis and poor prognosis. Furthermore, PUC is difficult to differentiate from signet ring cell carcinoma of the urinary bladder due to overlap in the clinical and morphological presentation [4]. In the present case, the initial diagnosis was signet ring cell carcinoma, thus we performed a radical cystectomy because of the poor prognosis. However, the subsequent pathological examination revealed infiltrating high-grade urothelial carcinoma with partial features of the plasmacytoid variant, thus we administered chemotherapy.

Most urological guidelines recommend adjuvant cisplatin-based chemotherapy as the therapy of choice in locally advanced bladder cancer. However, it has been reported that tumors with variant histology are associated with a higher risk of progression than conventional high-grade UC. Keck *et al.* reported that patients suffering from PUC have the worst clinical outcome regarding overall survival compared to conventional UC [7]. We searched for other cases of PUC that were treated with cystectomy and chemotherapy. Almost all cases were treated with cystectomy and adjuvant chemotherapy. All cases were at an advanced stage, and the chemotherapy consisted of methotrexate, vinblastine, doxorubicin and cisplatin (M-VAC) or GC. Cystectomy and adjuvant chemotherapy with GC was relatively effective for the management of PUC in terms of overall survival (Table 1). Furthermore, Kaimakliotis *et al.* reported that the management of PUC should be aggressive and that cystectomy should be performed at all stages [8]. They also reported that although it is unclear whether PUC is independently associated with a poor prognosis, the prognosis in PUC is poor due to the higher stage at the time of diagnosis [9].

**Table 1:** The cases of PUC that were treated with cystectomy and adjuvant chemotherapy

First author	TNM stage	Chemotherapy	Outcome	OS
Kohno [12]	T4N0M0	M-VAC×2	Survival	18 m
Kawashima [13]	T3aN0M0	M-VAC×2	Survival	11 m
Fritsche [14]	T4bN2M0	GC×4	Survival	16 m
	T4aN0M0	GC×5	Death	29 m
Soylu [15]	T2bNxM0	5FU+leucovorin	Death	18 m
Aldousari [16]	T3bN0M0	GC	Death	6 m
Hayashi [17]	T4bN0M0	GC×2	Death	9 m
Present case	T2bN0M0	GC×2	Survival	26 m

*PUC* invasive urothelial carcinoma, lymphoma-like/plasmacytoid variant, *TNM* tumor-node-metastasis, *OS* overall survival, *M-VAC* methotrexate, vinblastine, doxorubicin and cisplatin, *GC* gemcitabine and cisplatin, *5FU* 5-fluorouracil, *m* months

Recently, an anti-PD-L1 drug was found to have a rapid and ongoing response in patients with urothelial carcinoma in a phase 1 study. Interestingly, this drug showed higher efficacy in patients whose tumor-infiltrating cells showed high levels of *PD-L1* expression [10]. Boorjian *et al*. reported that higher PD-L1 expression in tumor cells was associated with the presence of advanced disease in patients with urothelial carcinoma and that it was correlated with a poor prognosis after radical cystectomy [11]. In our case, the tumor did not express PD-1 or PD-L1.

Although we predicted a poor prognosis for this variant, the tumor was successfully treated with a combination of radical cystectomy and adjuvant chemotherapy, and our patient has remained free of any sign of recurrence of bladder cancer for 2 years after the operation.

## Conclusions

We herein described the case of a patient with PUC controlled with chemotherapy following radical cystectomy.

## Consent

Written informed consent was obtained from the patient for publication of this case report and accompanying images. A copy of the written consent is available for review by the Editor-in-Chief of this journal.

## Abbreviations

CT: Computed tomography
GC: Gemcitabine and cisplatin
HPF: high-power field
MRI: Magnetic resonance imaging
M-VAC: Methotrexate, vinblastine, doxorubicin and cisplatin
PUC: Invasive urothelial carcinoma, lymphoma-like/plasmacytoid variant
TUR-Bt: Transurethral resection of a bladder tumor
UC: Urothelial carcinoma
